# Why Do People Exercise in Natural Environments? Norwegian Adults’ Motives for Nature-, Gym-, and Sports-Based Exercise

**DOI:** 10.3390/ijerph14040377

**Published:** 2017-04-04

**Authors:** Giovanna Calogiuri, Lewis R. Elliott

**Affiliations:** 1 Department of Dental Care and Public Health, Faculty of Public Health, Inland Norway University of Applied Sciences, Hamarveien 112, Elverum 2411, Norway; 2 European Centre for Environment and Human Health, University of Exeter Medical School, Knowledge Spa, Royal Cornwall Hospital, Truro, Cornwall TR1 3HD, UK; L.R.Elliott@exeter.ac.uk; 3 Psychology Applied to Health (PAtH), College House, University of Exeter Medical School, St Luke’s Campus, Exeter, Devon EX1 2LU, UK

**Keywords:** outdoor recreation, health promotion, physical activity, greenspace, sedentary, leisure time

## Abstract

Exercise in natural environments (“green exercise”) confers numerous health benefits, but little is known about why people engage in green exercise. This study examined the importance of nature experiences as a motive for physical activity and the motivational profile of people who engage in green exercise compared to gym- and sports-based exercise. Physical activity motives and typical times spent in different domains of physical activity were reported by 2168 Norwegian adults in a survey. Experiencing nature was generally rated as the second-most important physical activity motive, exceeded only by convenience motives, and it was especially important for older adults and those who engage in greater amounts of instrumental physical activity. Green exercisers reported stronger motives concerning convenience and experiencing nature, whereas gym- or sports-based exercisers reported stronger motives for physical health and sociability. The motives associated with different leisure-time exercise domains may assist in understanding optimal promotion of green exercise.

## 1. Introduction

Natural environments have emerged as useful settings for promoting physical activity because access to them has been consistently associated with moderate-to-vigorous physical activity attainment worldwide [1], although the link between natural environments, physical activity, and health is likely to be complex and it is not yet fully understood [2,3]. Any physical activity within natural environments is generally referred to as green exercise [4]. Studies show that green exercise is often of a health-enhancing intensity [5,6] and it has been associated with additive psychological benefits over physical activity in other types of environments, including reduction of psychophysiological stress and enhanced mental health [7]. Such positive psychological effects have also been shown to predict future engagement in physical activity [2,8]. It should be noted that the term *green* exercise not only refers to physical activity taking place in “green” spaces (i.e., environments dominated by the presence of grass and green foliage colours); an increasingly large body of evidence shows that physical activity in other natural environments, such as “blue” spaces (i.e., environments characterized by the presence of water) [9] and even “orange” spaces (i.e., landscapes dominated by fall foliage colour) [10], can also provide equivalent health-effects. Irrespectively on whether green exercise is actually performed in “green” spaces or other types of environments, promotion of green exercise is likely to relieve some of the health and economic burdens placed on society through inactivity as well as promote health in a broader sense. For instance, green exercise has been estimated to save society around £2.2 billion in the UK alone through welfare gains [11]. Knowing why people choose to engage in green exercise could inform promotional efforts in the future, but little research has been dedicated to this question to date.

Generally, different domains of leisure-time physical activity (LTPA) have been associated with different motivational profiles. For example, engaging in individual sports-based physical activity has been associated with enjoyment and mastery motives, while participating in fitness groups and other exercise has been associated with appearance-related motives [12,13]. One issue with these studies is that they conflate indoor and outdoor physical activity when the motives for each are likely to be different. Enjoying nature was reported as an important perceived benefit among visitors of natural parks [14]. Nature relatedness and feelings about nature were significant predictors of visiting nearby natural environments as well as engaging in high levels of green exercise [15,16]. The qualitative literature has also revealed the importance of nature experiences as a factor of motivation for green exercise. For example, leisure visits to UK parks were reported to be often motivated by opportunities to engage with natural qualities of the space as well as physical and cognitive restoration [17]. Similarly, enjoyment of engaging in outdoor activities and the sensory experience of nature were important meanings and values related to being active outdoors in a sample of middle-aged and older men living in a rural area of Norway [18]. However, the value of nature experiences as a motive for physical activity and, more generally, the motivational profile of those who engage in green exercise as a primary domain of LTPA remains little researched.

Systematic reviews of literature show that nature experiences can lead to positive psychophysiological states such as perceived stress relief and more positive states of wellbeing [7]. Studies also show that, when one exercises in the presence of nature, their focus of attention will be shifted towards the environment rather than towards internal feelings of fatigue, resulting in reduced perceived exertion [19,20]. As described in the model proposed by Calogiuri and Chroni [2], altogether, this can impact people’s intention to engage in physical activity and outdoor recreation, as well as help them sustain higher exercise intensities than they would sustain in other environments. Nature-related affective beliefs (e.g., feelings about nature) play an important role in this process, mediating the psychological effects of being exposed to nature and serving as an important motive to engage in green exercise [2,15]. However, the preceding motivation depends also on peoples’ environmental preferences and expected physical activity benefits [20,21], as well as on the characteristics of the individuals’ and their living environment [2,3].

### Present Study

In the present study, results from a national survey of Norwegian adults’ physical activity behaviours are utilised to discover the motives for different types of LTPA, including green exercise. Our research questions were:

1. What is the relative importance of nature experiences in relation to other physical activity motives among adults in Norway and what demographic characteristics are associated with them?
2. What physical activity motives are associated with participation in green exercise among adults in Norway and how do these differ from the motives associated with participation in other leisure-time physical activities?

## 2. Method

### 2.1. Respondents

In 2012, *Norsk Friluftsliv* (a Norwegian outdoor recreation organisation) commissioned a national survey which aimed to explore physical activity behaviours and motives among adult Norwegians, with particular emphasis on participation in green exercise. The web-based survey was administered by an external and independent market research company (Ipsos MMI) during October 2012. Invitations to participate were sent via email to 8620 individuals aged 18 or older, randomly selected from a panel of approximately 50,000 individuals who regularly participate in the company’s surveys. Participants each received a gift card as a reward for completing the survey. The sample was stratified by gender, age, and geographical area with the aim of recruiting a broad demographic representative of the Norwegian population. In total, 2168 responses were collected (response rate = 25%).

### 2.2. Measures

#### 2.2.1. Primary Domain of Leisure-Time Physical Activity

The outcome variable used in this study constituted the domain of moderate-to-vigorous intensity LTPA which the respondent undertook for the most time in a typical week. In the survey, the amount of time spent in moderate-to-vigorous physical activity in a typical week was measured with the item: “For how much time (hours and minutes) through the course of a regular week, do you engage in activities that increase your breathing or make you sweat?” Subsequently, respondents were asked to report how much of this time was spent undertaking a list of specific activities. Three of these activities could be considered leisure activities: “organised sports”, “exercising in the gym”, and “walking or exercising in parks, green spaces or other natural environments” (henceforth “green exercise”). For each respondent, each numeric response was converted into a percentage of the overall time spent engaged in LTPA. Each respondent was then assigned a primary domain of LTPA according to the type of leisure-time activity they engaged in for the highest percentage of time in a typical week. In all but 15 cases, this activity constituted over 50% of the overall time reported in the initial question. We recognise that, for example, sports can also take place in natural environments and therefore be considered green exercise. However, as participants were asked to recall sports participation and “walking or exercising in parks, green spaces or other natural environments” separately, we treat the latter as qualitatively distinct.

Other activities (“active transport to/from work or school”, “physical activity within school or work hours”, and “walking or exercising with a dog or other domestic animal”.) were listed in the questionnaire. Because of the more instrumental nature of these activities, they were not considered as LTPA and therefore not used for the primary outcome variable in the analysis. On the other hand, one may notice that the list of activity is clearly not comprehensive (e.g., activities such as running/walking/cycling in urban areas or exercising at home were not listed), as corroborated by the fact that for many respondents the overall amount of physical activity was greater than the sum of the time spent in the listed activity (more information about this is reported elsewhere [15]). This unaccounted physical activity is likely to contain different domains of LTPA; therefore, respondents for whom the majority of typical weekly LTPA was unaccounted for by the activities listed in the survey were excluded (*n* = 79) from further analysis.

Respondents who engaged in more than one LTPA for equivalent proportions of time were excluded (*n* = 113). In Norway, it is not uncommon for individuals to exercise their dog for intrinsic reasons (e.g., whilst running or sledding) as well as extrinsic reasons (to exercise the animal). Therefore, due to possible overlap with green exercise, those who reported “walking/exercising with dog or other domestic animal” as their primary domain of overall physical activity (*n* = 148) were excluded from final analysis. In total, 975 respondents’ primary domain was green exercise, 373 was gym-based exercise, and 200 was sports-based exercise. In addition to these three categories, a fourth category was assigned to 280 respondents who reported not engaging in any LTPA in a typical week.

#### 2.2.2. Motives for Physical Activity

In the survey, respondents were asked to rate the importance they assigned to 22 motives for engaging in physical activity generally on a scale from 1 (not important) to 4 (very important). A fifth option (does not apply to me) was not considered in the analysis. Two reasons, “to get fresh air” and “to experience nature”, were used to create a “nature experience” motive category (α = 0.81). It should be noted that although the motive “to get fresh air” might not necessarily relate to nature experiences, in this case it correlated well with the other motive “to experience nature”; furthermore, studies in the Norwegian population have previously reported that “fresh air” is often mentioned when Norwegians are asked to describe nature experiences [15,18]. Principal components analysis was used to cluster the other motives into superordinate groups. Components’ extraction was based on Eigenvalues greater than 1 [22], examination of scree plots [23], and factor loadings above 0.45 [24]. No motive loaded on more than one component. One motive (“to recover after sickness, pregnancy or injury”) was excluded on the basis of a low communality coefficient and factor loading. Five components were extracted. Briefly, these components were named “affective benefits” (α = 0.86), “convenience” (α = 0.68), “sociability” (α = 0.79), “long-term health” (α = 0.81), and “body-oriented benefits” (α = 0.74). Details of all six categories can be viewed in Table 1.

#### 2.2.3. Controls

A battery of demographic items was also recorded in the survey. Age and sex were controlled for as they have previously been associated with adults’ participation in different domains of LTPA [25]. Educational level has been positively associated with adults’ overall physical activity and the presence of young children in the household has been negatively associated with adults’ overall physical activity [26]; both of these were also controlled for in the analysis. Educational level was operationalised as two categories: those who had completed 13 years or less of education (i.e., up to the end of upper-secondary school in Norway) or those who had completed more than this, or who were currently studying (i.e., anyone in, or having completed, higher education, including university). The presence of young children in the household was operationalised as a binary variable. Participants’ home zip codes were recorded and from this we were able to identify whether they resided in an urban or rural location. This was controlled for because different patterns of leisure-time green exercise exist for urban and rural dwellers [5]. Lastly, participation in instrumental physical activities was controlled for. In addition to domains of leisure time physical activity, participants reported the time spent in a typical week engaged in activities such as “active transport to/from work or school”, “physical activity within school or work hours”, and “walking or exercising with a dog or other domestic animal”. The total time spent in these domains was calculated and used as a linear control variable in analyses. It was entered in minutes in the first analysis and in hours in the latter analysis to aid comprehension of the odds ratios (see Section 2.3).

### 2.3. Analytical Strategy

To address the first research question, a preliminary analysis was undertaken to determine what demographic characteristics were associated with “nature experience” and the other five superordinate physical activity motives. Using Wilks’ Lambda as the test statistic, multivariate analyses of variance (MANOVA) were undertaken where the six physical activity motives categories were set as dependent variables and sex, educational level, presence of young children in the household, and urban/rural residence were set as predictors in separate models. Age and instrumental physical activity were entered as continuous covariates in separate models. If a significant multivariate effect was observed, a univariate test (ANOVA) was performed to establish relationships between individual motives and the demographic characteristics.

A nominal logistic regression model was then developed to answer our second research question. This predicted respondents’ primary domain of LTPA from different motives for physical activity. To determine the extent to which different motives predicted green exercise, respondents whose primary domain of LTPA was green exercise were used as the reference category in comparison to the three other domains (gym-based, sports-based, and not typically engaged in LTPA). The primary domain of LTPA was regressed upon the five physical activity motives derived from the principal components analysis (entered as linear variables) as well as the other control variables. The “nature experience” motives category was subsequently added to the model in order to understand the contribution of these specific motives in predicting the respondents’ primary domain of LTPA.

## 3. Results

### 3.1. Sample Description

The sample was well balanced with respect to sex (50.4% males; 49.6% females), and age was normally distributed (median = 53.0 years). Most of the respondents had no responsibility for small children (71.1%), lived in urban areas (60.1%), and had a high educational level or were currently studying (63.5%). Importantly, the majority of respondents reported fairly high levels of overall physical activity (median = 180.00 min/week), which appear to be predominantly leisure-time physical activities. Among the instrumental domains of physical activity, “walking/exercising with a dog or other domestic animal” was the one which accounted for the greatest amount of overall physical activity (median = 120 min/week), with transport-related and occupational physical activity less so (median = 60 min/week for both domains). The different domains of LTPA were fairly equivalent in terms of weekly amounts of time the respondents spent in each of them (median = 120 min/week for all domains).

A comparison with national figures revealed that demographic characteristics such as sex, age, the residential location, and the prevalence of people having responsibility for small children, as well as overall physical activity levels, are in line with national statistics [27,28]. However, respondents’ education levels appeared slightly inflated compared to national figures. When excluding those who responded “currently studying”, only 10% of respondents in our sample had education attainment below upper secondary education, compared to 27% in the Norwegian population and 56% of respondents reported higher education attainment, compared to 36% in the Norwegian population [27].

### 3.2. Nature Experience and Other Physical Activity Motives

As shown in Table 2, “experiencing nature” was the second most important motive for physical activity in the sample, exceeded in importance only by “convenience”. “Affective” and “body-oriented” motives were also perceived as important, whereas “long-term health” and “sociability” motives were generally rated as less important. Results from the MANOVA can also be viewed in Table 2. There were significant multivariate effects for every demographic characteristic. “Experiencing nature” was especially important among women, older adults, and those who engage in greater amounts of instrumental PA during a regular week. Although the pattern of relative importance attributed to different motives remained relatively unchanged when observing each sex separately, females rated the importance of all motives significantly higher than males. Besides giving more importance to the experience of nature, older adults attributed more importance to long-term health motives, whereas younger respondents assigned more importance to affective benefits, and sociability motives. Respondents with higher education levels assigned significantly more importance to affective benefits and body-oriented benefits. Respondents with no young children in the household assigned greater importance to convenience motives than those with young children. Finally, besides giving more importance to the experience of nature, the respondents who engaged in more instrumental physical activity in a typical week also assigned more importance to affective benefits and convenience.

### 3.3. Motives for Green Exercise or Other Forms of Leisure-Time Physical Activity

The results of the nominal logistic regression can be viewed in Table 3. The model revealed distinct motivational profiles for respondents with different primary domains of LTPA. Firstly, in the model unadjusted for “nature experience”, higher convenience motives were significantly associated with a higher likelihood of having green exercise as a primary domain of LTPA compared to both gym-based and sports-based exercise. However, greater importance assigned to “body-oriented benefits” was associated with a significantly lower likelihood of green exercise compared with gym-based and sports-based exercise. Higher “long-term health” motives were associated with a higher likelihood, and “sociability” motives with a lower likelihood, of green exercise compared to sports-based exercise. Finally, higher importance assigned to “affective benefits” was associated with a higher likelihood of green exercise when compared with respondents undertaking no LTPA in a typical week. In short, participation in green exercise was associated with higher “convenience,” “affective benefits”, and (to a lesser extent) “long-term health” motives after adjustment for demographic variables and before adding “nature experience” into the model. Furthermore, older age was associated with a higher likelihood of green exercise compared with all other domains. Being male, of lower education, having young children in the household, and living in a rural area and engaging in more instrumental physical activity in a typical week were associated with a higher likelihood of green exercise compared to gym-based exercise. Having young children in the household was additionally associated with a higher likelihood of green exercise compared to sports-based exercise, whereas engaging in more instrumental physical activity in a typical week was additionally associated with a lower likelihood of green exercise compared to respondents who engage in no LTPA.

Most of these relationships remained after the inclusion of the “nature experience” motives into the model. However, long-term health motives were no longer associated with a higher likelihood of having green exercise as a primary domain of LTPA compared with sport-based exercise. The first new pattern to emerge was that higher “affective benefits” motives were now associated with a lower likelihood of green exercise compared with both gym-based and sports-based exercise, whereas they remained associated with higher likelihood of green exercise compared with those who engage in no LTPA. Higher motives for nature experience were, unsurprisingly, associated with a higher likelihood of green exercise compared to both gym-based and sports-based exercise, but not compared to those who engage in no LTPA. The associations with the sociodemographic variables remained also generally unchanged.

## 4. Discussion

### 4.1. Summary of Findings

The findings of this study show that experiencing nature is generally perceived as an important motive for physical activity in our sample, yielding the second-highest ratings of importance in our sample, preceded only by convenience motives. Nature experience motives were especially important among older adults and those who engage in greater amounts of instrumental physical activity during a regular week. Furthermore, distinct motivational profiles for respondents with different primary domains of LTPA were revealed: compared with those who mainly exercise in the gym or participate in sports, those who mainly engage in green exercise assigned more importance to nature experiences and convenience motives, and less importance to body-oriented and sociability motives.

### 4.2. The Importance of Nature Experiences as a Motive for Green Exercise

Norwegians are known for being generally fond of green exercise and outdoor recreation [29], and this could explain why nature experiences were attributed *such* high importance in our sample. Previous surveys in the Norwegian adult population have identified “preventing health problems” as the most important motive for physical activity [28], which is in line with other international studies [30]. These studies, however, did not include nature experiences (nor convenience) motives as an option for their respondents, and this could explain the differences with our findings. Consistent, in part, with previous cross-sectional literature, we found that experiencing nature was perceived as a more important motive for physical activity among females [31], older adults [32], and those who engage in greater amounts of instrumental physical activity during a regular week. Compared with males, females tended to assign greater importance to all motives for physical activity, therefore sex differences did not appear to be specifically related to nature experiences. Age presented quite a different pattern: the importance of experiencing nature increased with increasing age, while at the same time the importance of affective benefits and sociability motives decreased, suggesting that these motives are quite distinct from each other. The increased importance assigned to nature experiences in older adults is in line with the established literature. Studies have previously revealed that younger generations are less engaged with nature as compared with older generations [33]. Although such phenomena are not yet well explored in the physical activity domain, the findings observed in our sample support such findings in previous studies.

On the importance attributed to nature experiences, it was unsurprising that “convenience” was generally reported as the most important motive for physical activity: “lack of time” is known to be a very common barrier to physical activity [29,34] and two of the items in our “convenience” category, “That I can do it at any time, when it suits me best” and “That I can do it near home, school, workplace, etc.”, are clearly related to overcoming such a barrier. It is also unsurprising that this motive was perceived as more important among those who engaged in greater amounts of instrumental physical activity. Interestingly, these individuals also assigned greater importance to nature experience as a motive for physical activity. This supports, in part, the model proposed by Calogiuri and Chroni [2], according to which the presence of natural elements within people’s living environment can lead to positive affective responses that will in turn impact their physical activity levels, for example, by fostering instrumental forms of physical activity such as walking or biking to nearby destinations.

### 4.3. On the Motivational Profile of the Green Exercisers

Understandably, experiencing nature is confirmed to be an important correlate of green exercise. This is in line with quantitative and qualitative studies that have investigated the motives and values of individuals who visit natural environments and engage in outdoor recreation [14,15,16,17,18]. According to our findings, green exercisers are not driven by body-oriented motives in comparison to sports- and gym-based exercisers. In part, this can be explained by previous literature, as body image themes are closely intertwined with sports and fitness participation, at least in the media [35]. In contrast, motivation to engage in green exercise involves focusing on external factors such as the natural surroundings [36], rather than internal factors such as body image.

Another important motive that distinguished green exercisers from those who mainly engage in gym- and sports-based exercise was “convenience”. The importance of natural environments and urban green spaces to physical activity has been long advocated, based on the evidence that, if easily accessible and well maintained, natural environments can provide users with spaces where they can engage in physical activity free of charge and at times that better suit their daily schedules [2,37]. Interestingly, the item with the greatest factor loading within the category convenience was “That I can keep a comfortable pace, with no pressure from others”, suggesting that not only the economical-, accessibility-, and time-related convenience factors are important motives, but also the possibility of self-regulating exercise intensity according to personal preferences and comfort.

The association between affective motives and green exercise participation changed significantly after the motive “nature experiences” was added into the model. The relationship between green exercise and affective motives changed from being non-significant to being negative. However, such negative associations should not be interpreted as the green exercisers giving little importance to the affective benefits of physical activity in absolute terms. Exercise is known to provide psychological benefits independently of the environment it takes place in [38]. Moreover, peoples’ environmental preferences and expected physical activity benefits are important factors determining the extent to which one perceives natural environments as a suitable arena for their exercise [20,21]. Thus, our findings would indicate that those who assign greater importance to the affective benefits of physical activity, but are at the same time not motivated by experiencing nature, are more likely to exercise in the gym or participate in sport, especially if they believe that such environments can better provide better opportunities to pursue body-oriented and social benefits [20]. This interpretation is also supported by the fact that in the model adjusted for nature experience motives, negative associations with green exercise were found only in the comparisons with gym- and sports-based exercise, whereas the association remained positive when green exercise was compared with those who engage in no LTPA during an average week.

Males, those with lower education, those who have small children in the household, those who live in rural areas and those who engage in greater amounts of instrumental physical activity during a regular week were more likely to engage in green exercise than in gym-based exercise. No associations with demographic variables were observed when comparing green exercisers with those who engage in sports nor with those who do not engage in any LTPA during a regular week, suggesting that the demographic characteristics do not present major barriers to green exercise. These findings are in line, and at the same time, extend previous analyses in the same sample [22]. Attention should be given, however, to the association of green exercise with sex. We found that females were more likely to prefer gym-based exercise over green exercise. The relation between sex and use of natural environments for physical activity is not well understood, as the findings are mixed [2,3]. However, perceived safety is likely to be an important issue [3,39,40].

### 4.4. Implications

The information produced by this study could inform efforts in green exercise promotion, targeting specific groups of the population. For example, public health campaign messages could emphasise the fact that exercising in local parks or other nearby natural environments can provide pleasant nature experiences that make exercise more enjoyable, and at the same time people can do it when it suits them and according to their own pace, with no pressure from others. Given the near equivalent importance of nature experiences and convenience motives in the present study, one may assume that those who engage in no LTPA could be more easily persuaded to engage in green exercise rather than gym- or sport-based exercise. Therefore, such messages could especially target this group, for example, by leveraging on the fact that in natural environments people would encounter others who, like them, are not necessarily as motivated to do more structured forms of exercise. If framed in the correct way, these messages could persuade people to set specific plans of action for physical activity [41]; a known method of bridging the gap that sometimes exists between motivation and action [42].

On the other hand, messages that aim to persuade gym- or sport-exercisers to integrate their exercise routines with some green exercise should emphasize the health benefits of exercising in nature, hinting, for example, to the fact that one can sustain higher exercise intensities [19], as well as the fact that natural environments provide opportunities to meet people and engage in social activities. Message could also be designed to target younger adults, who appear to be less engaged with green exercise in comparison to older adults. Promotional efforts should not, however, be limited to public health campaigns. For example, promotional efforts to encourage women to engage in more green exercise should also include infrastructural interventions that aim to improve the perceived safety of local natural environments, for example, by improving lightning and designing spaces to offer high levels of prospect (clear field of vision), while at the same time evoking lower levels of refuge (places where potential risks may be concealed) [39].

### 4.5. Limitations

This is the first study to determine the motivational profile of adults who undertake green exercise as their primary domain of LTPA. However, due to the cross-sectional nature of this study, the analysis is subject to a number of limitations. Firstly, the analysis assumes that participants accurately recall the time spent in different domains of leisure-time physical activity. Since the perception of elapsed time can often be longer when engaged in green exercise compared to other forms of exercise [9], it is plausible that participants recalled a greater duration of green exercise than was actually the case. Nonetheless, there is evidence that all forms of physical activity can lead to lengthened perceptions of time [43], so there may be no reason to suggest that recall of time in green exercise was systematically misremembered more than any other domain.

Secondly, and more pertinently to the present study, the analyses represent associations between time spent in different LTPA domains and motives for physical activity generally. This means that we cannot ascribe any individual motive to any particular episode of physical activity. For example, just because ratings of “convenience” motives for physical activity are generally associated with more participation in green exercise, this does not mean that any one episode of green exercise was motivated by its convenience. Future research may wish to interrogate datasets that have the ability to associate specific active visits to natural environments with motives for that specific visit; the UK’s Monitor of Engagement with the Natural Environment survey [44] is one such dataset which allows this possibility, albeit in a non-Scandinavian context. Furthermore, we recognise that participation in green exercise (or gym- or sports-based exercise) may simply be habitual behaviour, and not motivated by any specific motivation that was captured in the survey. Previous research has identified this to be the case in a sample of recreational runners based in the UK [45] using a qualitative, ethnographic approach, and similar research deserves further attention in the future as an understudied aspect of behaviour formation.

Thirdly, the data from the survey determined how we clustered together different motives. While we believe a principle components analysis was the fairest way to group the individual motives, it also means that other conceptually related motives may have been separated. For example, self-determination theory, a psychological theory of motivation, posits that feelings of autonomy over one’s behaviour—the perception that one is competent enough to perform a behaviour—and feelings of relatedness or personal connection, converge to support the development and enactment of motivations [46]. The motives “that I can do it at any time which suits me,” and, “I think I have to” are both clearly related to autonomous motivations for physical activity (the latter obviously referring to non-autonomy), however, in this analysis both are clustered under different superordinate motivational constructs. Since systematic reviews have previously demonstrated consistent positive relationships between autonomous forms of motivation and exercise participation [47], clustering motives based on a psychological theory such as self-determination theory may have been a useful avenue for investigating whether green exercise is associated with more autonomous forms of motivation. Future research may want to cluster motives according to theories of motivation in order to test hypotheses about proposed pathways of such theories.

Lastly, our measures of green exercise might have overlapped with other forms of leisure-time or instrumental physical activity, or even under-estimated the extent to which respondents are exposed to nature whilst engaging in different activities. For instance, we could not account for whether the respondents were exposed to nature when commuting to work/school, walking the dog, participating in sports, etc. These arguably more incidental forms of green exercise deserve greater attention in future research.

## 5. Conclusions

In a large sample of Norwegian adults, participation in green exercise was associated with motives concerning convenience and the opportunity to experience nature. These represent distinct motivational profiles from those who spend more time engaged with gym- or sports-based exercise. Nature experience was also an important motivator for older adults and those who engaged in greater amounts of instrumental physical activity. Future research could investigate whether motives for green exercise are more intrinsic or extrinsic by investigating combinations of motivational factors that are in line with psychological theories of motivation. Nonetheless, the data presented here could help to inform how to motivate different sub-populations to engage in green exercise in the future.

## Figures and Tables

**Table 1:** Grouped motives for physical activity according to results from principal components analysis.

Superordinate Group and Included Items ^a^	N ^c^	Eigenvalues	α
Nature experience **^b^** To experience nature To get fresh air	2130	-	0.81
Affective benefits I experience mental wellbeing when I’m in good shape I experience physical wellbeing when I’m in good shape To relax, reduce stress Because I enjoy it To get excitement, challenges It gives me better self-confidence	2137	6.15	0.86
Convenience That I can keep a comfortable pace, with no pressure from others That the activity is free or reasonably cheap That I can do it at any time, when it suits me best That I can do it near home, school, workplace, etc.	2146	1.79	0.68
Sociability That I can be together with others Being with my friends	2144	1.61	0.79
Long-term health To reduce sick-leave from work/school To have a long work-life To be independent, active and healthy when I’ll retire	2119	1.32	0.81
Body-oriented benefits To keep/reduce my bodyweight I think I have to To get physical strength To prevent health problems	2139	1.05	0.74

**^a^** Included items are ranked by factor loading; **^b^** This category was created “ad-hoc”, as the two included items are closely related to green exercise; **^c^** Different sample sizes are the result of excluding respondents who answered that the individual motive “did not apply” to them.

**Table 2:** Results from a MANOVA analysis examining perceived importance attributed to the different motives across demographic groups in the sample (*n* = 2096) ^a^.

	Motives for Physical Activity (M ± SD)					
Variable	Nature Experience	Affective Beliefs	Convenience	Sociability	Long-Term Health	Body-Oriented Beliefs
**Overall Sample**	3.16 ± 0.75	3.02 ± 0.66	3.26 ± 0.54	2.52 ± 0.78	2.93 ± 0.82	3.00 ± 0.62
**Sex**						
Male	3.04 ± 0.76	2.93 ± 0.67	3.17 ± 0.56	2.42 ± 0.77	2.81 ± 0.82	2.86 ± 0.62
Female	3.28 ± 0.71	3.11 ± 0.63	3.34 ± 0.49	2.62 ± 0.78	3.06 ± 0.79	3.15 ± 0.58
MANOVA: F_(6, 2089)_ = 27.40 ***						
*ANOVA: F_(1, 2094)_ = …*	60.03 ***	36.60 ***	54.46 ***	32.50 ***	51.91 ***	119.71 ***
**Age**						
(continuous)	t = 6.56	t = −2.37	t = 1.13	t = −4.62	t = 10.28	t = −1.35
MANOVA: F_(6, 2089)_ = 52.00 ***						
*ANOVA: F_(1, 2094)_ = …*	42.97 ***	5.61 *	1.27	21.35 ***	105.77 ***	1.83
**Education**						
Lower education	3.14 ± 0.75	2.95 ± 0.66	3.25 ± 0.57	2.56 ± 0.77	2.92 ± 0.82	2.96 ± 0.65
Higher education	3.17 ± 0.74	3.06 ± 0.65	3.26 ± 0.51	2.50 ± 0.78	2.94 ± 0.81	3.03 ± 0.59
MANOVA: F_(6, 2089)_ = 4.43 ***						
*ANOVA: F_(1, 2094)_ = …*	0.76	11.91 **	0.02	2.88	0.54	6.42 *
**Young Children at Home**						
No	3.17 ± 0.75	3.02 ± 0.66	3.27 ± 0.53	2.51 ± 0.80	2.95 ± 0.83	2.99 ± 0.63
Yes	3.12 ± 0.73	3.03 ± 0.64	3.21 ± 0.54	2.54 ± 0.74	2.90 ± 0.78	3.04 ± 0.59
MANOVA: F_(6, 2089)_ = 3.11 **						
*ANOVA: F_(1, 2094)_ = …*	2.07	0.09	6.89 **	0.55	1.27	2.30
**Residential Location**						
Urban area	3.13 ± 0.75	3.02 ± 0.65	3.24 ± 0.53	2.54 ± 0.77	2.90 ± 0.82	3.02 ± 0.59
Rural area	3.20 ± 0.74	3.02 ± 0.66	3.28 ± 0.55	2.48 ± 0.79	2.97 ± 0.81	2.98 ± 0.66
MANOVA: F_(6, 2089)_ = 3.68 **						
*ANOVA: F_(1, 2094)_ = …*	3.67	0.00	2.25	3.30	3.72	1.86
**Instrumental physical activity**						
(continuous)	t = 3.69	t = 2.58	t = 3.41	t = 1.02	t = 1.65	t = −1.04
MANOVA: F_(6, 2089)_ = 5.22 ***						
*ANOVA: F_(1, 2094)_ = …*	13.59 ***	6.68 **	11.60 **	1.03	2.73	1.09

* p < 0.05; ** p < 0.01; *** p < 0.001 ^a^ The reduced sample size is due to the exclusion of respondents who answered that any of the individual motives items “did not apply” to them.

**Table 3:** Nominal logistic regression modelling the relationship of favourite leisure-time physical activity (LTPA) with different motives in adult Norwegians, after controlling for selected background (*n* = 1761 ^a^).

Predictor	Primary Domain of LTPA—OR (95% CI)		
	Exercise in the Gym vs. Green Exercise	Participate in Sports vs. Green Exercise	Not Engage in LTPA vs. Green Exercise
***Model I (Pseudo R^2^: Cox and Snell = 27%; Nagelkerke = 30%)***			
Affective benefits	1.12 (0.85–1.47)	0.98 (0.69–1.39)	2.51 (1.89–3.33) ***
Convenience	3.10 (2.35–4.08) ***	3.94 (2.84–5.48) ***	1.18 (0.87–1.60)
Sociability	1.16 (0.97–1.40)	0.44 (0.34–0.57) ***	1.07 (0.86–1.34)
Long-term health	0.96 (0.78–1.18)	1.31 (1.02–1.69) *	1.07 (0.85–1.34)
Body-oriented benefits	0.25 (0.18–0.34) ***	0.61 (0.42–0.87) **	1.18 (0.87–1.60)
Age	1.04 (1.03–1.05) ***	1.04 (1.03–1.05) ***	1.04 (1.03–1.05) ***
Sex			
Male	1.33 (1.01–1.74) *	0.95 (0.68–1.34)	1.11 (0.81–1.45)
Female = ref			
Education			
Lower education	1.51 (1.13–2.02) **	1.17 (0.83–1.65)	1.00 (0.73–1.37)
Higher education = ref			
Having small children			
No	0.64 (0.48–0.86) **	0.64 (0.45–0.93) *	0.99 (0.71–1.37)
Yes = ref			
Centrality			
Urban area	0.71 (0.54–0.94) *	1.25 (0.89–1.75)	1.01 (0.74–1.39)
Rural area = ref			
Overall instrumental PA	1.12 (1.03 1.22) **	1.06 (0.97 1.17)	0.93 (0.88 0.97) **
***Model II (Pseudo R^2^: Cox and Snell = 30%; Nagelkerke = 34%)***			
Nature experience	2.51 (1.96-3.21) ***	2.60 (1.93–3.50) ***	1.26 (0.95–1.66)
Affective benefits	0.64 (0.47–0.89) **	0.58 (0.39–0.86) **	2.19 (1.58–3.03) ***
Convenience	2.59 (1.95–3.45) ***	3.14 (2.23–4.41) ***	1.14 (0.84–1.55)
Sociability	1.08 (0.89–1.30)	0.41 (0.32–0.53) ***	1.06 (0.85–1.33)
Long-term health	0.90 (0.72–1.11)	1.19 (0.92–1.54)	1.05 (0.84–1.32)
Body-oriented benefits	0.25 (0.18–0.35) ***	0.62 (0.43–0.90) *	1.18 (0.87–1.61)
Age	1.03 (1.02–1.04) ***	1.03 (1.02–1.05) ***	1.04 (1.02–1.05) ***
Sex			
Male	1.48 (1.12–1.96) **	1.04 (0.74–1.47)	1.14 (0.83-1.56)
Female = ref			
Education			
Lower education	1.50 (1.11–2.01) **	1.16 (0.82–1.64)	1.00 (0.73–1.37)
Higher education = ref			
Having small children			
No	0.65 (0.48–0.89) **	0.66 (0.45–0.95) *	0.97 (0.70–1.36)
Yes = ref			
Centrality			
Urban area	0.73 (0.55–0.97) *	1.28 (0.91–1.81)	1.02 (0.74–1.40)
Rural area = ref			
Overall instrumental PA	1.11 (1.02 1.20) *	1.06 (0.96 1.16)	0.92 (0.88 0.97) **

* *p* < 0.05; ** *p* < 0.01; *** *p* < 0.001 ^a^ Reduced sample size is the result of (i) excluding respondents who answered “do not apply” to any individual motive (*n* = 67); (ii) excluding respondents who spent equal amounts of time in a typical week engaged in more than one LTPA domain (*n* = 113); (iii) excluding respondents who reported that the majority of their typical moderate-to-vigorous physical activity in a typical week was unaccounted for by the specific types of activity explored in the survey (*n* = 79), and; (iv) excluding respondents who reported exercising a dog or other domestic animal (*n* = 148, see Section 2.2.1).
